# Supplementary figures and images for: CNP blocks mitochondrial depolarization and inhibits SARS-CoV-2 replication in vitro and in vivo
Source: PLoS Pathog. 2023 Dec 20;19(12):e1011870. doi: 10.1371/journal.ppat.1011870 (PMC10766180; doi:10.1371/journal.ppat.1011870)

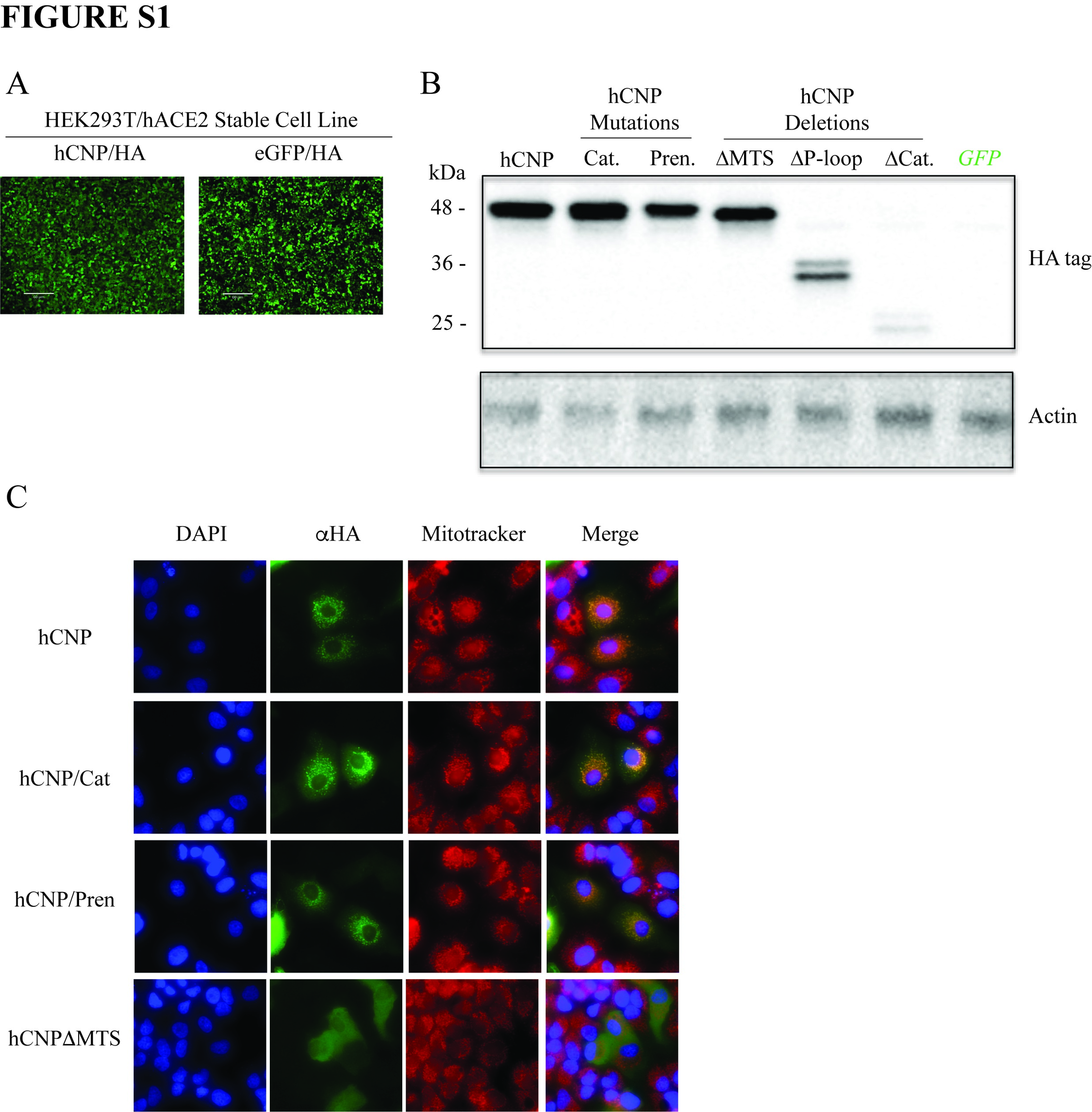

Supplement: S1 Fig — (a) HA-tag staining of HEK293T/hACE2 cells transduced with lentivirus vectors expressing either HA-tagged CNP or HA-tagged eGFP and maintained under selection media. (b) Western blots from HEK293T/hACE2 cells transfected with CNP mutation and deletion plasmid constructs stained for HA-tag or Actin controls. (c) Colocalization of HA-tagged CNP constructs in A549/hACE2 cells with mitochondrial staining by mitotracker. Merged images show DAPI, anti-HA and mitotracker colocalization. Image is representative of triplicate samples. (TIF) [file ppat.1011870.s001.tif]

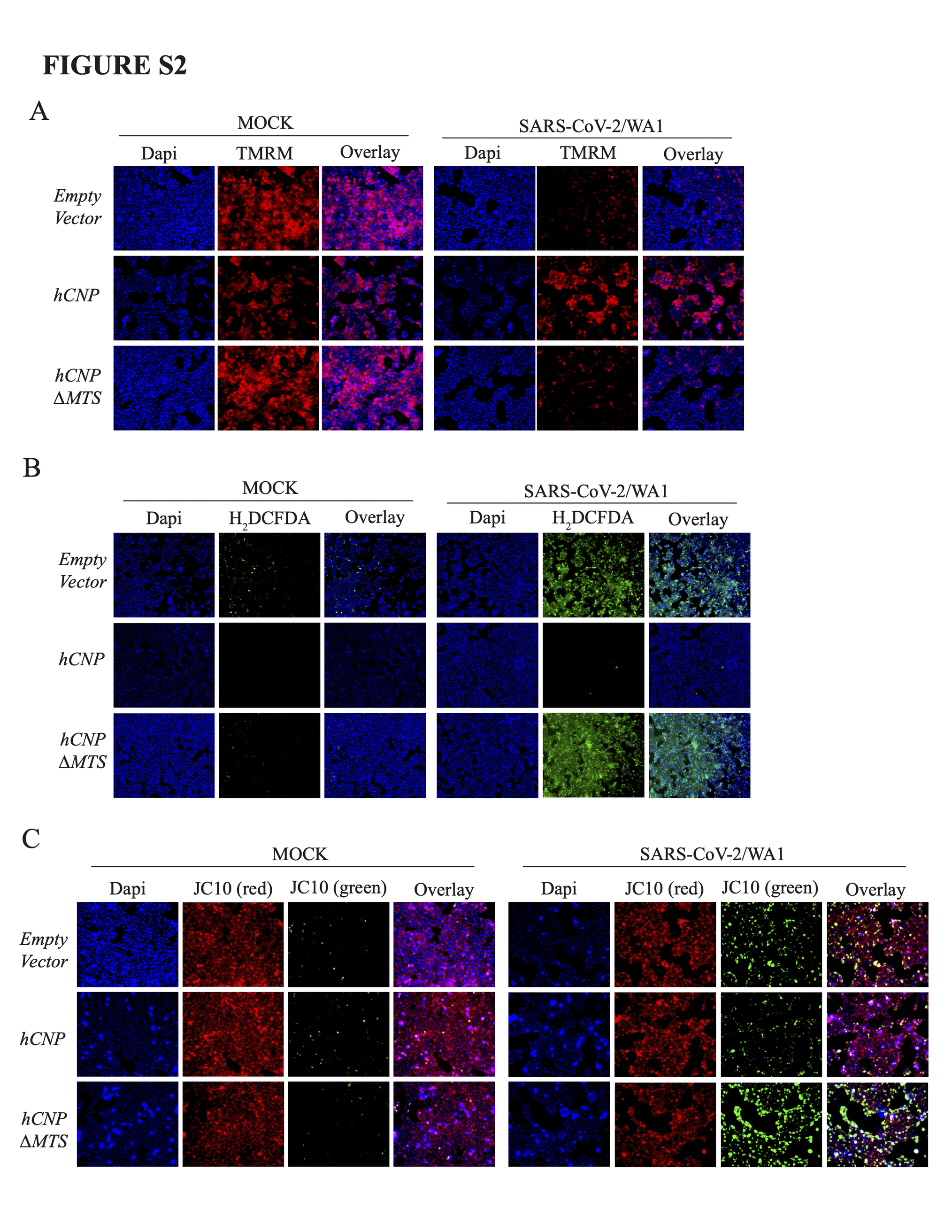

Supplement: S2 Fig — Images correspond to Fig 3. HEK293T/hACE2 cells were infected with SARS-CoV-2/WA1 or MOCK infected 24-hours following transfection with indicated plasmids. At 6-hours post-infection, mitochondrial function was assessed for (a) opening of the mPTP with TMRM staining, (b) reactive oxygen species staining with H2DCFDA, or (c) mitochondrial depolarization with JC10 staining. (TIF) [file ppat.1011870.s002.tif]

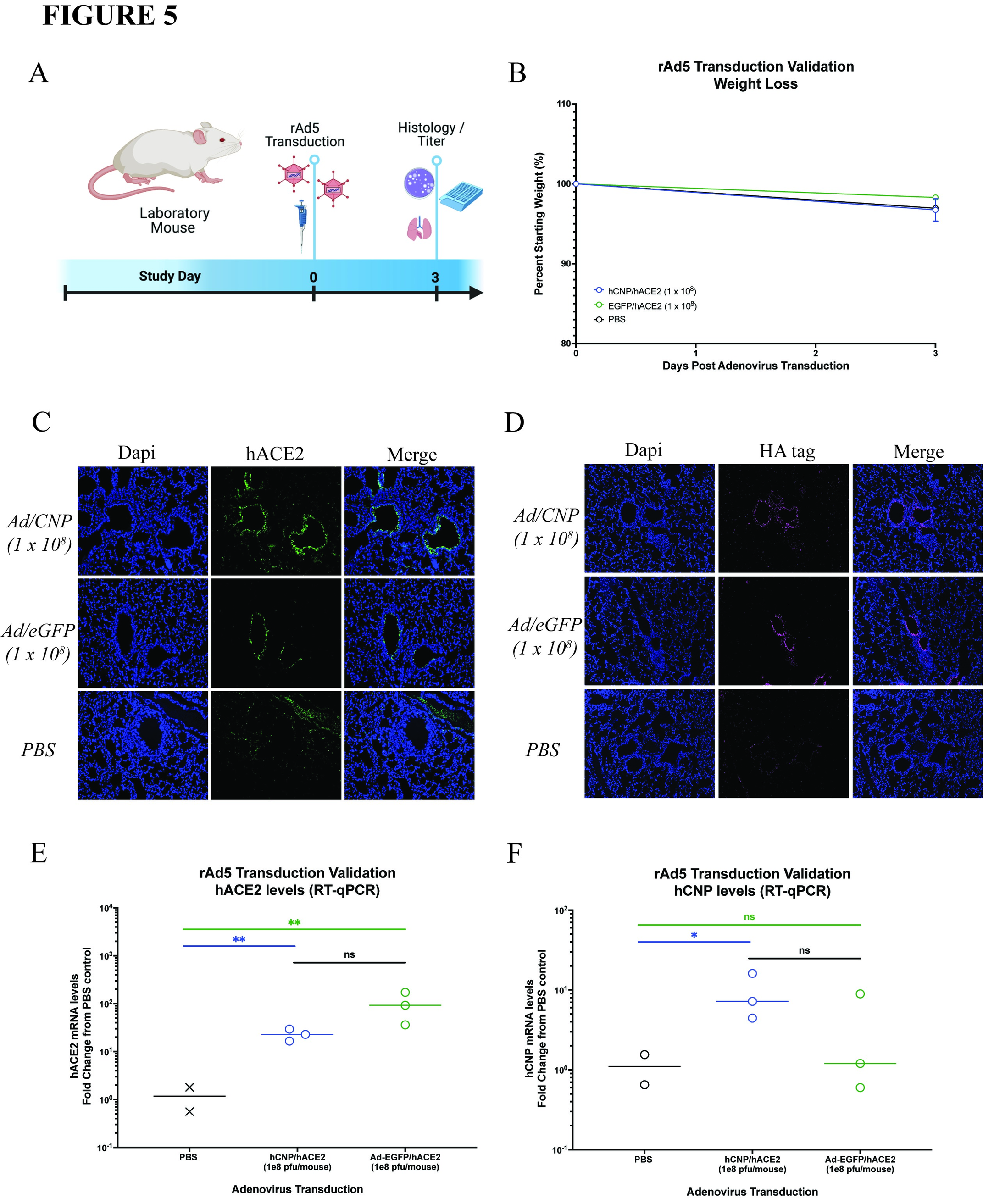

Supplement: S3 Fig — (a) Groups of mice were transduced intranasally with rAd5 vectors (1 x 10^8^ viral particles per mouse) dually expressing hACE2 and either HA-tagged CNP or HA-tagged eGFP. Image created using BioRender. (b) Weight changes were determined at 3 days post-transduction, plotted as the group mean with error bars indicating the ±SD. c-d Lungs were collected at 3-days post-transduction and co-stained by immunofluorescence staining for (c) nucleus (blue) and hACE2 (green) or (d) nucleus (blue) and HA-tag (purple). e-f RT-qPCR was performed for lung homogenates collected at 3-days post-transduction for (e) hACE2 and (f) CNP. *p ≤ 0.1, **p ≤ 0.01. (TIF) [file ppat.1011870.s003.tif]
