# Supplementary material for: CNP blocks mitochondrial depolarization and inhibits SARS-CoV-2 replication in vitro and in vivo
Source: PLoS Pathog. 2023 Dec 20;19(12):e1011870. doi: 10.1371/journal.ppat.1011870 (PMC10766180; doi:10.1371/journal.ppat.1011870)
Supplement: S1 Table — (DOCX) [file ppat.1011870.s004.docx]

**Table S1: RT-qPCR primers.**

| **Gene Target** | **Forward (5’ to 3’) or**  **Company (Cat#)** | **Reverse (5’ to 3’) or**  **Company (Cat#)** | **Detection**  **Method** |
| --- | --- | --- | --- |
| sgRNA (SARS2 N) | ctcttgtagatctgttctctaaacgaac | ggtccaccaaacgtaatgcg | SYBR Green |
| gRNA (SARS2 RdRp) | Integrated DNA Technologies (*10006860*) | Integrated DNA Technologies (10006881) | SYBR Green |
| Human ACE2 | ------- Integrated DNA Technologies (*Hs01085333_m1*) ------- | | SYBR Green |
| Human CNP | ----- Integrated DNA Technologies (*Hs.PT.58.27238178*) ----- | | FAM Probe |
| Mouse GAPDH | --------- Integrated DNA Technologies (*Mm.PT.39a.1*) ---------- | | SYBR Green |
| Human GAPDH | ----- Integrated DNA Technologies (*Hs.PT.39a.22214836*) ----- | | SYBR Green |

Abbreviations: “sgRNA”: subgenomic RNA; “gRNA”: genomic RNA; “ACE2”: human angiotensin converting enzyme II; “CNP”: human 2’,3’ cyclic-nucleotide 3’, phosphodiesterase; “GAPDH”: glyceraldehyde-3-phosphate dehydrogenase;
