# Supplementary material for: CNP blocks mitochondrial depolarization and inhibits SARS-CoV-2 replication in vitro and in vivo
Source: PLoS Pathog. 2023 Dec 20;19(12):e1011870. doi: 10.1371/journal.ppat.1011870 (PMC10766180; doi:10.1371/journal.ppat.1011870)
Supplement: S2 Table — (DOCX) [file ppat.1011870.s005.docx]

**Table S2: Infusion cloning primers.**

| **Gene Target**  **(mutations)** | **Frag.** | **Forward (5’ to 3’)** | **Reverse (5’ to 3’)** |
| --- | --- | --- | --- |
| hCNP | N/A | tcattttggcaaagaattcaccatgaacagaggcttctcc | atcataagggtacatcccgggtatgatggtgcagga |
| hCNP-CatMut  *(H251A, T253A,*  *H330A, T332A)* | 1 | tcattttggcaaagaattcaccatgaacagaggcttctcc | aacttggtagcgcaagccagcacgcctgg |
|  | 2 | ttgcgctaccaagttttgtgactacg | cagccgagagcgatagcggcgcggctccc |
|  | 3 | tatcgctctcggctgtgca | atcataagggtacatcccgggtatgatggtgcagga |
| hCNP-PrenMut  (*C418A*) | N/A | tcattttggcaaagaattcaccatgaacagaggcttctcc | aagggtacatcccgggtatgatggtggcggactgcaaggc |
| hCNP∆MTS  (*∆1-20*) | N/A | ttttggcaaagaattcatgtcatcctcaggggcca | atcataagggtacatcccgggtatgatggtgcagga |
| hCNP∆Ploop  (*∆36-148*) | N/A | agtttcccgagcccaagacggcgtgg | tgggctcgggaaactgcagctcaggc |
| hCNP∆Cat  (∆*185-399*) | N/A | agaaggacaaaggcaaacctgtgccc | tgcctttgtccttctccagcccaggc |

Abbreviations: “hCNP”: human 2’,3’ cyclic-nucleotide 3’, phosphodiesterase; “CatMut”: catalytic site mutation; “PrenMut”: prenylation site mutation; “MTS”, mitochondrial targeting sequence; “Cat”: catalytic domain;
