# Supplementary material for: CNP blocks mitochondrial depolarization and inhibits SARS-CoV-2 replication in vitro and in vivo
Source: PLoS Pathog. 2023 Dec 20;19(12):e1011870. doi: 10.1371/journal.ppat.1011870 (PMC10766180; doi:10.1371/journal.ppat.1011870)
Supplement: S3 Table — (DOCX) [file ppat.1011870.s006.docx]

**Table S3: Antibodies for *in vitro* Western Blot and Immunofluorescent staining**

| **Assay** | **Target**  **(Detection)** | **Antibodies (animal, cat#, supplier)** | **Staining**  **Conc.** |
| --- | --- | --- | --- |
| **Western Blot** | **HA-tag**  (HRP) | 1°: Rabbit; H6908 (Sigma) | 0.6 µg/mL |
|  |  | 2°: Goat α-rabbit HRP; 31460 (Invitrogen) | 0.4 µg/mL |
|  | **SARS2 S**  (HRP) | 1°: Rabbit; NB100-56578 (Novus) | 1.0 µg/mL |
|  |  | 2°: Goat α-rabbit HRP; 31460 (Invitrogen) | 0.4 µg/mL |
|  | **SARS2 N**  (HRP) | 1°: Rabbit; 40143-R004 (Sino Biologicals) | 1.0 µg/mL |
|  |  | 2°: Goat α-rabbit HRP; 31460 (Invitrogen) | 0.4 µg/mL |
|  | **Actin**  (HRP) | 1°: Mouse; Ab7817 (Abcam) | 1.0 µg/mL |
|  |  | 2°: Goat α-mouse HRP; 31430 (Invitrogen) | 0.2 µg/mL |
| **IFA**  **(*in vitro)*** | **HA-tag**  (alexa488) | 1°: Chicken; Ab9111 (Abcam) | 1.0 µg/mL |
|  |  | 2°: Goat α-chicken 488; A11039 (Invitrogen) | 1.0 µg/mL |
|  | **HA-tag**  (alexa647) | 1°: Chicken, Ab9111 (Abcam) | 1.0 µg/mL |
|  |  | 2°: Goat α-chicken 647; A21449 (Invitrogen) | 1.0 µg/mL |
|  | **SARS2 S**  (alexa594) | 1°: Rabbit; NB100-56578 (Novus) | 1.0 µg/mL |
|  |  | 2°: Donkey α-Rabbit 594; Ab150064 (Abcam) | 1.0 µg/mL |
|  | **SARS2 N**  (alexa594) | 1°: Rabbit; 40143-R004 (Sino Biologicals) | 1.0 µg/mL |
|  |  | 2°: Donkey α-Rabbit 594; Ab150064 (Abcam) | 1.0 µg/mL |
|  | **ERGIC53**  (alexa647) | 1°: Mouse; ABS300-100 (Enzo) | 10.0 µg/mL |
|  |  | 2°: Goat α-mouse 647; A21235 (Invitrogen) | 1.0 µg/mL |
| **IFA (histology)** | **hACE2**  (alexa488) | 1°: Goat; AF933 (R&D) | 2.0 µg/mL |
|  |  | 2°: Rabbit α-Goat 488; Ab11078 (Abcam) | 2.0 µg/mL |
|  | **HA-tag**  (alexa647) | 1°: Chicken, Ab9111 (Abcam) | 10.0 µg/mL |
|  |  | 2°: Goat α-chicken 647; A21449 (Invitrogen) | 2.0 µg/mL |

Abbreviations: “Conc.”: concentration; “HRP”: horse radish peroxidase; “IFA”: immunofluorescence assay; “SARS2”: SARS-CoV-2; “N”: nucleocapsid protein; “S”: spike protein; “hACE2”: human ACE2;
